# Nerve Transfers and Adjunct Procedures for Restoration of Shoulder External Rotation in Obstetrical Brachial Plexus Palsy: Long-Term Outcomes and Review of the Literature

**DOI:** 10.3390/jcm14207415

**Published:** 2025-10-20

**Authors:** Filippo M. Sénès, Annalisa Valore, Chiara Arrigoni, Maria Grazia Calevo, Nunzio Catena

**Affiliations:** 1 Hand Surgery Department, San Giuseppe Hospital, IRCSS MultiMedica, 20123 Milan, Italy; filipposenes@fastwebnet.it; 2 Hand Surgery Department, Ospedale P. Pederzoli, 37019 Peschiera del Garda, Italy; annalisavalore@hotmail.it; 3 Hand Surgery and Reconstructive Microsurgery, IRCCS Istituto Giannina Gaslini, 15147 Genoa, Italy; chiara.arrigoni@libero.it; 4 Epidemiology and Biostatistic Unit, Scientific Direction, IRCCS Istituto Giannina Gaslini, 15147 Genoa, Italy; mariagraziacalevo@gaslini.org

**Keywords:** nerve transfers, obstetrical brachial plexus palsy, shoulder medial contracture

## Abstract

**Background**: Obstetrical brachial plexus palsy (OBPP) often results in medial shoulder contracture, with limited abduction and external rotation due to muscle imbalance and joint deformities. Late nerve transfers, such as spinal accessory nerve (SAN) to suprascapular nerve (SSN) transfer, combined with soft tissue release, represent a therapeutic option for shoulder reanimation in children presenting after infancy. **Methods**: 56 children treated between 2007 and 2019 have been evaluated. Inclusion criteria were as follows: age at time of surgery > 9 months, no primary reconstruction of the brachial plexus, late presentation, two years of follow-up. Patients were divided into groups based on age (<18 months vs. >18 months) and procedures: SAN to SSN transfer associated with subscapularis release (58.9%), SAN to SSN transfer associated with coracohumeral ligament release (17.9%), isolated SAN to SSN (12.5%), multiple nerve transfer (10.7%). Universal Mallet Grading Score was applied. A review of literature on the topic published on Pub Med up to December 2024 was associated with the retrospective analysis of clinical data. **Results**: At 2 years 84% of patients achieved Mallet Scores > 3, with progressive improvement up to 5 years. No significant differences were observed between age groups or type of palsy. Coracohumeral ligament release demonstrated comparable effectiveness to subscapularis release with fewer complications. Secondary surgical interventions were required in 30% of cases, mainly in those undergoing multiple nerve transfers. **Conclusions**: SAN to SSN transfer is a reliable and effective procedure for restoring shoulder external rotation. Coracohumeral ligament release provides a minimally invasive means to improve passive range of motion while preserving internal rotation muscle integrity. These combined interventions may reduce the need for more invasive secondary surgeries.

## 1. Introduction

Medial contracture of the shoulder, along with limited abduction and external rotation, affects many children with obstetrical brachial plexus palsy (OBPP). Several factors contribute to this condition, including mechanical imbalance between the recovery of internal and external muscles, dysregulation of protein pathways [1,2,3] and predominant atrophy of the subscapularis muscle. The most advanced manifestation of this complex dysfunction is glenohumeral joint dysplasia (GHD).

The prevalence of GHD is considerable: passive range of motion (ROM) limitation exceeding 10° occurs in up to 56% of cases; limitations greater than 20° and 30° are observed in 31% and 17% of patients, respectively. Osseous deformities affect approximately 33% of patients, ranging from glenoid retroversion to posterior humeral subluxation [4,5].

The degree of glenoid retroversion has been closely correlated with subscapularis muscle atrophy, as reported by Freedman et al. [6,7,8,9]. Conversely, Waters et al. found that posterior humeral subluxation correlates with abnormalities in all rotator cuff muscles [10]. Notably, Hogendoorn et al. identified an inverse relationship between the severity of plexus injury (classified by Narakas Group) and shoulder incongruity: patients with total brachial plexus palsy, involving both internal and external rotators, were less likely to develop bony deformities than those with upper trunk palsy (C5–C6/C7), due to absence of muscle imbalance [4].

Additional upper limb deformities may develop, including elbow flexion contracture, forearm supination, ulnar deviation of the wrist and various patterns of finger paralysis, which further compromise overall limb function.

The pathogenesis of shoulder internal contracture is complex, involving both neurological and mechanical factors. Consequently, therapeutic and surgical interventions aimed at controlling or preventing this sequela may not always achieve satisfactory outcomes.

Primary brachial plexus repair with nerve grafts remains the first line treatment for infants with complete palsy or non-recoverable upper palsy (C5–C6/C7). However, timing is crucial, as reconstructive attempts beyond 10 months of age, when fibrosis become extensive, are generally unsuccessful [11,12,13].

Secondary orthopedic procedures, including muscle tendon lengthening or release, functional muscle-tendon transfers and corrective osteotomies, are typically reserved for established deformities in older children.

Neurotization, widely used as an adjunct to primary repair when donor roots are unavailable, represents the most viable option for reanimating selective functions in children over one year old. Indications include late presentation, atypical or incomplete functional recovery or failed primary repair. The timing of nerve transfers in OBPP remains flexible for at least two reasons: first, muscle denervation is rarely complete in OBPP (unlike in adults), allowing some axons to remain functional and compensate by “adopting” others; second, children possess significant neuroplasticity and regenerative potential. These factors support the use of nerve transfers to restore selective functions and extend the period for achieving motor control well into childhood.

Our experience with late nerve transfers for OBPP began in 2005 at the Gaslini Institute. Since then, various techniques employing donor nerves, such as the spinal accessory nerve, intercostal nerves and branches of the ulnar or radial nerves, have been utilized in single or multiple transfers, as previously detailed by the senior author [14].

This retrospective study aims to evaluate the outcomes of external rotator muscle reanimation via spinal accessory nerve (SAN) to suprascapular nerve (SSN) transfer in 56 patients with long-term follow-up ranging from two to ten years. To deal with shoulder contracture, soft tissue procedures such as subscapularis muscle release or coracohumeral ligament release were combined.

Our intention for pursuing the study was to verify that nerve coaptation could prevent skeletal deformities, muscle imbalance, joint contractures and physeal arrest, thereby reducing the need for secondary procedures (e.g., latissimus dorsi transfer or external rotation osteotomy).

## 2. Materials and Methods

A retrospective analysis was conducted on 56 pediatric patients (43 males, 13 females) treated for obstetrical brachial plexus palsy (OBPP) between 2007 and 2019. The patients’ ages at the time of surgery ranged from 9 months to 6 years (mean age 21 months). Twenty-nine patients (51.7%) were younger than 18 months at the time of intervention. All cases were unilateral, with a slight right-side predominance (53.7%). The distribution of OBPP types was as follows: 36 patients (64.3%) with C5–C6 involvement and 20 patients (35.7%) with C5–C6–C7 involvement. No cases of total brachial plexus palsy were included.

Inclusion criteria were:

- Age > 9 months at the time of surgery
- Late presentation without indication for primary nerve repair
- Upper trunk palsy with atypical or incomplete functional recovery during early infancy
- Minimum postoperative follow-up of 2 years

Patients with previous brachial plexus reconstruction using nerve grafts were excluded.

Patients were categorized into two age-based cohorts: <18 months and >18 months, to evaluate age related differences in surgical outcomes. Based on the surgical technique performed, patients were also divided into four subgroups:

1. SAN to SSN transfer with subscapularis release (n 33, 58.9%)
2. SAN to SSN transfer with coracohumeral release (n 10, 17.9%)
3. Isolated SAN to SSN transfer (n 7, 12.5%)
4. Multiple nerve transfers (n 6, 10.7%)

Data are also presented in Table 1.

Shoulder contracture was defined as passive external rotation ≤ 20° from the sagittal plane, with the arm adducted and elbow flexed to 90°. Due to high prevalence of passive and active external rotation limitations, nerve transfers were combined with palliative soft tissue procedures in 76.8% of cases.

From 2007 to 2016, the posterior subscapularis release was the preferred technique. From 2016 onward, the anterior coracohumeral ligament release was adopted as a less invasive alternative. In all cases, a pre-molded thoraco-brachial cast was applied postoperatively for 3 weeks to protect the neurorrhaphy and maintain the achieved external rotation. The immobilization position included 90° shoulder abduction, 90° elbow flexion and forearm supination.

Functional outcomes were assessed using the Modified Mallet Score at baseline, 6 months, 1 year and 2 years. Additional assessments were conducted at 3, 5, 7 and 10 years in patients with extended follow-up.

Descriptive statistics were computed for the entire cohort. Continuous variables are presented as mean ± standard deviation (SD), median and range; categorical variables are expressed as absolute numbers and percentages. The distribution of continuous data was assessed using the Kolmogorov–Smirnov test.

For group comparisons, the Mann–Whitney U test and Wilcoxon signed-rank test were employed for continuous variables, while the Chi-square test of Fisher’s exact test was used for categorical variables, depending on expected frequencies. A two-tailed *p*-value of <0.05 was considered statistically significant. All statistical analyses were performed using SPSS (version 29) for Windows (SPSS Inc., Chicago, IL, USA).

We conducted a retrospective analysis of clinical data based on the published literature on the subject in PubMed until December 2024.

## 3. Results

The primary analysis focused on functional outcomes two years postoperatively and the longitudinal progression of scores over time, as assessed by the Modified Mallet Score (MS). At the two years follow-up, most patients achieved either Grade 3 (32%) ore Grade 4 (52%). Two patients (4%) showed limited improvement (MS 2), whereas six patients (12%) reached optimal outcomes (MS 5).

The trend in functional recovery continued to improve up to 5 years postoperatively, with no cases scoring below MS 3. Specifically, 18.8% of patients scored MS 3, 46.9% scored MS 4 and 34.4% reached MS 5, indicating good to excellent outcomes. Clinical progression is detailed in Table 2.

Paired analysis (baseline vs. final follow-up) revealed statistically significant improvement in Mallet scores over time. The mean score increased to 4.16 ± 0.72 at 5 years (*p* < 0.0001). Scores remained stable thereafter, with means of 4.17 ± 0.79 at 7 years and 4.15 ± 0.80 at 10 years (*p* < 0.0001). These findings are illustrated in Table 3.

When comparing outcomes by age at surgery using a threshold of 18 months, no statistically significant difference was observed. However, patients older than 18 months at the time of surgery demonstrated slightly higher mean functional scores (Table 4).

No significant differences were in relation to the type of surgical procedure or the type of palsy (C5–C6 vs. C5–C7). However, patients in Group 1 (SAN to SSN + subscapularis release) and Group 4 (SAN to SSN + multiple nerve transfers) showed a higher incidence of requiring secondary procedures compared to patients in Groups 2 and 3 (SAN to SSN ± coraco-humeral ligament release). Detailed data are presented in Table 5 and Table 6.

As shown in Table 7, no statistically significant association was found between the need for secondary (palliative) procedures and either the type of initial nerve transfer or the brachial plexus palsy pattern. However, patients in the SAN + subscapularis and SAN + other transfer groups appeared more likely to undergo secondary surgery compared to those who received SAN only transfers, regardless of coracohumeral release.

## 4. Discussion

This study was conducted at a specialized referral center for obstetrical brachial plexus palsy in Italy. Since the 1990s, the surgical team has been committed to developing and refining techniques in plexus reconstruction and nerve transfers with the goal of improving functional outcomes in affected children.

We focused specifically on shoulder internal contracture and evaluated the outcomes of spinal accessory nerve (SAN) to suprascapular nerve (SSN) transfer in a well-defined cohort of 56 patients with long term follow-up ranging from 2 to 10 years. The majority (76.8%) underwent concomitant soft tissue procedures, either subscapularis muscle release (SCMR) or coraco-humeral ligament release (CHLR), to address mechanical restrictions.

Previous literature, including seminal works by Birch and Gilbert, identified internal rotation contracture as a principal factor limiting shoulder function, primarily due to coraco-humeral ligament retraction and muscle imbalances involving the subscapularis, pectoralis major and teres major muscles [15,16]. Early intervention, ideally before 4 years of age, has been advocated to prevent progressive bone deformities and improve passive range of motion. Our findings corroborate the importance of early functional restoration, as shoulder dysfunction impairs the entire upper limb kinematic chain, limiting essential activities such as hand-to-mouth and hand-to-head movements.

Historically, subscapularis muscle detachment was employed to improve external rotation but was associated with intraoperative bleeding risks. Since 2016, our practice shifted towards CHLR, a minimally invasive technique originally used for adult frozen shoulder, which offers comparable correction of external rotation (up to 60°) with reduced surgical morbidity, shorter operative time and preservation of internal rotator muscles. This approach aligns with recent prospective studies demonstrating no significant differences between SCMR and CHLR, with a preference for the latter due to its safety profile.

C. Sarac et al. compared posterior SSR and anterior CHLR in a prospective study involving 102 children, concluding that the outcomes were almost equivalent, with no statistically significant difference, ultimately favoring CHLR as the preferred technique [17]. Other studies have described procedures such as anterior capsular release or CHLR performed in combination with subscapularis tenotomy/lengthening or latissimus dorsi muscle transfer [18,19].

In our opinion, sacrificing the subscapularis muscle is unnecessary and leads to weakening of the shoulder not only in internal rotation, but also in abduction and elevation—planes in which the subscapularis acts as a prime mover. Therefore, CHLR is currently our first choice, almost always combined with SAN-SSN neurotization. Consequently, SSR is now limited to refractory and older cases.

Coaptation of the distal spinal accessory nerve (SAN) to the suprascapular nerve (SSN) is a feasible and effective technique, and it has now become a standardized method for shoulder reanimation in late-presenting OBPP cases. Several high-quality studies are available to support or contrast our findings. Van Ouwerkerk in 2006 [20] studied the effects of SAN-SSN transfer in 54 patients (aged 6–84 months), reporting that 72% experienced short recovery time (within 4 months postoperatively), with at least 20° of improved active external rotation and a progressive improvement curve at 8–12 and 24 months, reaching a final functional level of 40°. Subscapularis tendon lengthening was combined in 14 cases.

Interestingly, the outcomes were not significantly influenced by age (before or after 12/18 months). The authors reached two important conclusions:

1. Spinatus muscle atrophy is reversible, with almost complete recovery of muscle mass at two years postoperatively, as shown in MRI follow-up.
2. Results were poorer when SAN to SSN transfer was part of primary graft reconstruction in six patients (mean age 5 months), possibly due to the young age and the negative impact of extensive plexus grafting on clinical recovery [20].

Late primary nerve surgery has also been investigated by the TOBI Study Group (MC Daly et al., 2019 [21]), following encouraging reports published in recent years [22,23,24,25,26].

Surgery was performed between 9 and 22 months of age, and the study population was heterogeneous (76% Narakas I–II, 24% Narakas III–IV). Nerve transfers (n = 20) and nerve grafts (n = 12) yielded similar outcomes. However, delayed selective SAN to SSN transfer (n = 10) in upper trunk lesions was especially noteworthy, with Active Movement Scale (AMS) scores improving from 0 preoperatively to 3 at one year and 6 at two years postoperatively [21].

These findings align with other reports on the effectiveness of SAN-SSN transfer in shoulder reanimation. IM Abelmaksoud et al. conducted a prospective study on 20 patients and observed a favorable distribution of final AMS scores: grade 5 in 9.45%, grade 6 in 4.20%, and full grade 7 in 2.10%. Conversely, patient age showed a statistically significant negative correlation with active range of motion: younger patients (<16 months) experienced quicker reinnervation. Significant differences were found at six months post-op for external rotation and at 12 months for abduction [27].

The role of age, however, remains unresolved. In our study, no statistically significant differences were observed between patients younger and older than 18 months (both groups were similarly represented), although the Mallet index appeared slightly higher in the older group.

This raises the question of whether it may be advisable to delay nerve transfers in selected cases—but we do not provide any recommendations at this stage.

In our series, shoulder reanimation was successful in 83% of patients (MS3) at six months postoperatively, with progressive improvement over two years distributed as follows: 4% MS2, 32% MS3, 52% MS4, and 12% MS5. These values improved further at five-year follow-up, with the majority reaching Mallet grades 4 (46.9%) and 5 (34.4%). After this period, outcomes tended to stabilize. As expected, patients with C5–C6 palsy showed better results than those with C5–C7 involvement.

Another focus of our study is the frequency of secondary procedures following nerve transfers.

The preventive role of reinnervation in avoiding skeletal dysplasia—which may result in permanent functional impairment—is not a new concept. Schaakxs in 2009 [28] reported on 65 patients with OBPP who underwent SAN to SSN transfer: 37.8% achieved 90° of active external rotation at 36 months, and 71.5% demonstrated good active functional ROM (“hand-to-head”) between 60° and 90°. Notably, 7 patients showed spontaneous regression of shoulder dysplasia postoperatively.

Although nerve transfers for external rotation restoration are widely accepted, adverse effects or complications are rarely addressed [29].

Is there still a role for palliative procedures? We believe that muscle transfers or humeral external rotation osteotomy—depending on the patient’s age—still play an important role in the long-term management of OBPP. In our series, 17 patients benefited from latissimus dorsi muscle transfer or skeletal correction, which further improved external rotation, even after nerve transfers. We observed occasional scapular winging due to partial trapezius weakness but no cases requiring additional surgical stabilization.

Despite the success of nerve transfers, palliative procedures such as muscle transfers and corrective osteotomies remain important for long term management. In our series, 17 patients underwent such secondary interventions, resulting in additional improvements in external rotation. Ipsilateral and contralateral lower trapezius transfer, commonly used in adult brachial plexus injuries, is gaining recognition as a valuable option in pediatric OBPP cases [30].

### Limitations of the Study

This study has some limitations that should be considered when interpreting the findings:

- Retrospective Design: The analysis was retrospective, which may introduce selection bias and limit the ability to establish causal relationships.
- The absence of a non-surgical or alternative-surgical comparator group: the lack of a comparator group can limit causal attribution. However, our goal was to report outcomes of SAN→SSN transfer as a standard, widely accepted approach in late-presenting OBPP. We agree that future prospective studies with comparator groups would better clarify its relative efficacy.
- No formal statistical comparisons (e.g., *p*-values or confidence intervals) between subgroups: We acknowledge the limited statistical power due to small subgroup sizes. Our primary aim was to assess longitudinal functional improvement in the overall cohort. Subgroup analyses by age, surgical technique, and type of palsy (Table 4, Table 5, Table 6 and Table 7) were performed, but no statistically significant differences emerged.
- Procedural heterogeneity: the inclusion of multiple surgical techniques can introduce potential confounding factors. However, our cohort reflects real-world surgical practice, where soft tissue releases are tailored to each patient’s presentation. Despite procedural differences, no statistically significant outcome variation was observed among subgroups (Table 5), though we acknowledge the limited size of some groups.
- Lack of Advanced Imaging: CT or MRI scans were not performed to evaluate the grade of glenohumeral dysplasia (GHD) before and after nerve transfer and soft tissue procedures, potentially affecting the accuracy of anatomical assessment. Limitations of the Mallet Score: The Mallet scoring system does not allow for precise evaluation of isolated external rotation recovery, which may shadow some functional improvements following intervention. We selected it due to its ease of application in pediatric population and its value in assessing overall shoulder function, although future studies could benefit from incorporating more granular outcome measures.

## 5. Conclusions

The spinal accessory nerve to suprascapular nerve transfer is a reliable and effective procedure for reanimating external rotator muscles in children with OBPP-related shoulder contracture. Concomitant soft tissue release, preferably coracohumeral ligament release, should be performed to address mechanical limitations and optimize range of motion. This combined surgical approach, addressing both neurological and mechanical factors, offers significant functional improvement.

We recommend coraco-humeral ligament release over subscapularis muscle release due to its minimally invasive nature, lower risk profile, muscle preservation and reduced recurrence.

Although some patients required further interventions to optimize outcomes, our findings demonstrate that nerve transfers significantly improve upper limb function in infancy and early childhood, laying the groundwork for future surgical refinements if needed.

## Figures and Tables

**Table 1:** Patient Demographics and Surgical Group Distribution.

Demographics and Clinical Data	N (%)
Gender, Males	37 (66.1)
Side, Right	31 (55.4)
Palsy C5–C6	36 (64.3)
Palsy C5–C6–C7	20 (35.7)
**Surgical Group**	**N (%)**
Group I—SAN + SSN + SC release	33 (58.9)
Group II—SAN + SSN + CO release	10 (17.9)
Group III—SAN + SSN only	7 (12.5)
Group IV—Multiple nerve transfers	6 (10.7)

Abbreviations: SAN = Spinal accessory nerve; SSN = Suprascapular nerve; SC = Subscapular nerve; CO = Coraco-Humeral.

**Table 2:** Longitudinal Outcomes Based on Mallet Score.

Mallet Score	Pre-op	6 mo	1 yr	2 yr	3 yr	5 yr	7 yr	10 yr	Final
Score 1	3 (5.4%)	–	–	–	–	–	–	–	–
Score 2	53 (94.6%)	8 (15.1%)	3 (5.7%)	2 (4.0%)	1 (2.4%)	–	–	–	–
Score 3	–	44 (83%)	20 (37.7%)	16 (32%)	11 (26.2%)	6 (18.8%)	4 (22.2%)	3 (23.1%)	2 (20%)
Score 4	–	1 (1.9%)	27 (50.9%)	26 (52%)	19 (45.2%)	15 (46.9%)	7 (38.9%)	5 (38.5%)	6 (60%)
Score 5	–	–	3 (5.7%)	6 (12%)	11 (26.2%)	11 (34.4%)	7 (38.9%)	5 (38.5%)	2 (20%)

Note: Values are reported as N (%). Mallet scores range from 1 (poor function) to 5 (normal function). Follow-up was conducted at multiple intervals post-surgery.

**Table 3:** Paired Comparison of Preoperative and Postoperative Mallet Scores.

Comparison	N	Pre-op (Mean ± SD)	Post-op (Mean ± SD)	*p*-Value
Pre-op vs. 6 months	53	1.96 ± 0.19	2.87 ± 0.39	<0.0001
Pre-op vs. 1 year	53	1.96 ± 0.19	3.57 ± 0.69	<0.0001
Pre-op vs. 2 years	50	1.96 ± 0.20	3.72 ± 0.73	<0.0001
Pre-op vs. 3 years	42	1.98 ± 0.15	3.95 ± 0.79	<0.0001
Pre-op vs. 5 years	32	1.97 ± 0.18	4.16 ± 0.72	<0.0001
Pre-op vs. 7 years	18	2.00 ± 0.00	4.17 ± 0.79	<0.0001
Pre-op vs. 10 years	13	2.00 ± 0.00	4.15 ± 0.80	0.001
Pre-op vs. Final	10	1.90 ± 0.32	4.00 ± 0.67	0.004

Note: All comparisons showed statistically significant improvements in Mallet scores over time until approximately 4 years of age. Thereafter, scores remain stable.

**Table 4:** Effect of Age at Surgery on Functional Outcomes: Comparison ≤18 vs. >18 Months.

Time Point	≤18 Months (Mean ± SD)	>18 Months (Mean ± SD)	*p*-Value
Pre-op	1.89 ± 0.32	2.00 ± 0.00	0.07
6 months	2.96 ± 0.39	2.81 ± 0.40	0.33
1 year	3.54 ± 0.71	3.59 ± 0.69	0.90
2 years	3.67 ± 0.70	3.77 ± 0.76	0.64
3 years	3.84 ± 0.76	4.04 ± 0.82	0.34
5 years	4.00 ± 0.73	4.31 ± 0.70	0.27
7 years	3.86 ± 0.90	4.36 ± 0.67	0.25
10 years	4.00 ± 0.82	4.33 ± 0.82	0.53
Final	4.17 ± 0.75	3.75 ± 0.50	0.48

Note: No statistically significant differences were observed between the two age groups.

**Table 5:** Functional Outcomes Stratified by Surgical Technique.

Time Point	Group I (SAN + SSN + SC)	Group II (SAN + SSN + CO)	Group III (SAN + SSN Only)	Group IV (Multiple Transfers)
Pre-op	1.97 ± 0.17	1.90 ± 0.32	1.86 ± 0.38	2.00 ± 0.00
6 months	2.82 ± 0.46	3.00 ± 0.00	3.00 ± 0.00	2.83 ± 0.41
1 year	3.52 ± 0.75	3.89 ± 0.60	3.40 ± 0.55	3.50 ± 0.55
2 years	3.65 ± 0.75	3.88 ± 0.75	4.00 ± 1.00	3.67 ± 0.52
3 years	3.88 ± 0.83	4.17 ± 0.75	4.40 ± 0.89	3.67 ± 0.52
5 years	4.09 ± 0.73	4.50 ± 0.71	4.50 ± 1.00	4.00 ± 0.00
7 years	4.07 ± 0.83	–	5.00 ± 0.00	4.00 ± 0.00
10 years	4.00 ± 0.87	–	5.00 ± 0.00	4.00 ± 0.00
Final	4.00 ± 0.76	–	–	4.00 ± 0.00

Note: no statistical differences were identified in relation to the type of surgical procedures.

**Table 6:** Comparison of Outcomes by Type of Brachial Plexus Palsy (C5–C6 vs. C5–C6–C7).

Time Point	C5–C6 (n = 36) Mean ± SD	C5–C6–C7 (n = 20) Mean ± SD	*p*-Value
Pre-op	1.97 ± 0.17	1.90 ± 0.31	0.25
6 months	2.91 ± 0.38	2.79 ± 0.42	0.29
1 year	3.59 ± 0.70	3.53 ± 0.70	0.71
2 years	3.78 ± 0.75	3.61 ± 0.70	0.48
3 years	4.00 ± 0.86	3.85 ± 0.55	0.54
5 years	4.24 ± 0.78	3.86 ± 0.38	0.21
7 years	4.20 ± 0.86	4.00 ± 0.00	0.65
10 years	4.18 ± 0.87	4.00 ± 0.00	0.77
Final	4.00 ± 0.71	4.00 ± 0.71	1.00

Note: no statistical differences were identified in relation to the type of palsy.

**Table 7:** Association Between Secondary (Palliative) Procedures and Initial Surgical Approach or Palsy Pattern.

Variable	No Palliative Surgery (n = 39)	Underwent Palliative Surgery (n = 17)	*p*-Value
**Primary Nerve Transfer**			
SAN + Subscapularis (SC)	21 (63.6%)	12 (36.4%)	0.55
SAN + Coraco-Humeral (CO)	8 (80.0%)	2 (20.0%)	–
SAN only	6 (85.7%)	1 (14.3%)	–
SAN + Other Transfers	4 (66.7%)	2 (33.3%)	–
**Palsy Type**			
C5–C6	25 (69.4%)	11 (30.6%)	1.00
C5–C6–C7	14 (70.0%)	6 (30.0%)	–

Abbreviations: SAN = Accessory Spinal Nerve; SC = Subscapularis muscle; CO = Coraco-Humeral ligament. Note: No statistically significant association was found between the need for secondary (palliative) procedures and either the type of initial nerve transfer or the brachial plexus palsy pattern. However, patients in the *SAN + subscapularis* and *SAN + other transfer* groups appeared more likely to require secondary procedures compared to those who underwent *SAN-only* transfers, irrespective of coraco-humeral release.

## Data Availability

All the data can be requested from the authors.
